# Variants of Severe Acute Respiratory Syndrome Coronavirus 2 (SARS-CoV-2) and Vaccine Effectiveness

**DOI:** 10.3390/vaccines10101751

**Published:** 2022-10-19

**Authors:** SubbaRao V. Tulimilli, Siva Dallavalasa, Chaithanya G. Basavaraju, Vinay Kumar Rao, Prashanth Chikkahonnaiah, SubbaRao V. Madhunapantula, Ravindra P. Veeranna

**Affiliations:** 1 Center of Excellence in Molecular Biology and Regenerative Medicine (CEMR) Laboratory (DST-FIST Supported Center), Department of Biochemistry (DST-FIST Supported Department), JSS Medical College, JSS Academy of Higher Education & Research (JSS AHER), Mysuru 570004, Karnataka, India; 2 Department of Medical Genetics, JSS Medical College & Hospital, JSS Academy of Higher Education & Research (JSS AHER), Mysore 570015, Karnataka, India; 3 Department of Pulmonary Medicine, Mysore Medical College and Research Institute, Mysuru 570001, Karnataka, India; 4 Special Interest Group in Cancer Biology and Cancer Stem Cells (SIG-CBCSC), JSS Medical College, JSS Academy of Higher Education & Research (JSS AHER), Mysuru 570004, Karnataka, India; 5 Department of Biochemistry, Council of Scientific and Industrial Research (CSIR)-Central Food Technological Research Institute (CFTRI), Mysuru 570020, Karnataka, India

**Keywords:** COVID-19, SARS-CoV-2, delta, Omicron, Variants Being Monitored, Variants of Concern, variants of interest, Variants of High Consequence, vaccine efficacy

## Abstract

The incidence and death toll due to SARS-CoV-2 infection varied time-to-time; and depended on several factors, including severity (viral load), immune status, age, gender, vaccination status, and presence of comorbidities. The RNA genome of SARS-CoV-2 has mutated and produced several variants, which were classified by the SARS-CoV-2 Interagency Group (SIG) into four major categories. The first category; “Variant Being Monitored (VBM)”, consists of Alpha (B.1.1.7), Beta (B.1.351), Gamma (P.1), Delta (B.1.617.2), Epsilon (B.1.427, B.1.429), Eta (B.1.525), Iota (B.1.526), Kappa (B.1.617.1), Mu (B.1.621), and Zeta (P.2); the second category; “Variants of Concern” consists of Omicron (B.1.1.529). The third and fourth categories include “Variants of Interest (VOI)”, and “Variants of High Consequence (VOHC)”, respectively, and contain no variants classified currently under these categories. The surge in VBM and VOC poses a significant threat to public health globally as they exhibit altered virulence, transmissibility, diagnostic or therapeutic escape, and the ability to evade the host immune response. Studies have shown that certain mutations increase the infectivity and pathogenicity of the virus as demonstrated in the case of SARS-CoV-2, the Omicron variant. It is reported that the Omicron variant has >60 mutations with at least 30 mutations in the Spike protein (“S” protein) and 15 mutations in the receptor-binding domain (RBD), resulting in rapid attachment to target cells and immune evasion. The spread of VBM and VOCs has affected the actual protective efficacy of the first-generation vaccines (ChAdOx1, Ad26.COV2.S, NVX-CoV2373, BNT162b2). Currently, the data on the effectiveness of existing vaccines against newer variants of SARS-CoV-2 are very scanty; hence additional studies are immediately warranted. To this end, recent studies have initiated investigations to elucidate the structural features of crucial proteins of SARS-CoV-2 variants and their involvement in pathogenesis. In addition, intense research is in progress to develop better preventive and therapeutic strategies to halt the spread of COVID-19 caused by variants. This review summarizes the structure and life cycle of SARS-CoV-2, provides background information on several variants of SARS-CoV-2 and mutations associated with these variants, and reviews recent studies on the safety and efficacy of major vaccines/vaccine candidates approved against SARS-CoV-2, and its variants.

## 1. Introduction

Severe Acute Respiratory Syndrome Coronavirus-2 (SARS-CoV-2) is a virus which surfaced in late 2019 in the city of Wuhan, China. The virus has spread across the globe quickly, leading to a pandemic situation causing acute respiratory disease known as “coronavirus disease 2019” (COVID-19) [1]. As of 21 August 2022, the ongoing pandemic has registered more than 600 million confirmed cases and 6.4 million deaths reported by the WHO [2]. The top five countries with highest numbers of cases reported are the United States of America (85,007,630), India (43,309,473), Brazil (31,611,769), France (29,164,805), and Germany (27,211,896). The top five countries with the highest number of reported deaths are the United States of America (1,002,946), Brazil (668,693), India (524,873), the Russian Federation (380,517), and Mexico (325,271) (https://covid19.who.int/ (accessed on 21 June 2022).

Although mutations are a characteristic feature of all viruses, the rate of mutations in RNA viruses is much higher than that of DNA viruses [3,4]. A study showed that the mutability magnitude of RNA viruses is five-fold compared to DNA viruses [5]. Although SARS-CoV-2, has a low sequence diversity compared to many other RNA viruses, the genetic recombination due to 3′-to-5′ exoribonuclease (nsp14) activity has produced numerous variants of SARS-CoV-2 [6,7]. In the process of evolution, a few viruses may mutate, and new variants are expected to develop over time as variants of the original virus. These variants can vary among themselves by one or more mutations [8]. A few newer variants may appear and disappear randomly and only a few may persist for a longer time [9]. The natural selection decides the destiny of the new variants [7,10]. Some mutations can negatively affect the viral replication, transmission or immunity escape, which consequently reduces the abundance of virus.

Mutations in the virus genome have an important influence on virulence, transmissibility, pathogenesis, diagnosing, treatment, as well as vaccine development [11,12]. Since “S” protein of SARS-CoV-2 is one of the primary targets in vaccine design strategy, mutations in the S region can reduce the efficacy of vaccines against the virus. Hence, mutations generated in the emerging variants should be monitored in vaccinated and non-vaccinated positive cases. Several SARS-CoV-2 variants have already been reported and documented worldwide during the COVID-19 pandemic [13]. To expand synchronization among Center for Disease Control and Prevention (CDC), National Institutes of Health (NIH), Biomedical Advanced Research Development Authority (BARDA), Food and Drug Administration (FDA) and Department of Defense (DoD), US Department of Health and Human Services (HHS) has established a SARS-CoV-2 Interagency Group (SIG). The main aim of this group is to rapidly characterize emerging variants and actively monitor their potential impact on critical SARS-CoV-2 counter-measures, including vaccines, therapeutics, and diagnostics [14]. CDC, in association with SIG has established a classification system for SARS-CoV-2 variants based on the threat level they pose to the public health and classified variants into four major types. The first type is “Variants Being Monitored (VBM)”, and the second type is “Variants of Concern (VOC)”. Variants of Interest and Variants of High Consequence (VOHC) are the third and fourth types, respectively [14]. The established nomenclature systems for naming and tracking SARS-CoV-2 lineages by GISAID, Nextstrain, and Pango are currently in use among scientific community [15]. The Nextstrain nomenclature system is based on the diversity of SARS-CoV-2 patterns and label clades that can persist for at least several months; and have a significant geographic spread [15]. The Pango lineage nomenclature is focused on the epidemiological event(s) such as the introduction of a virus into a distinct geographic area with evidence of onward spread [16]. WHO along with group of scientists (WHO Virus Evolution Working Group, the WHO COVID-19 reference laboratory network), representatives (GISAID, Nextstrain, Pango) and experts (virological, microbial nomenclature) from several countries and agencies, developed an easy-to-pronounce and non-stigmatizing labels for variants to assist discussion among public [15]. WHO expert group has recommended the use of Greek Alphabets, i.e., Alpha, Beta, Gamma, Delta (classified as previous VOC), Omicron (classified as current VOC) and Epsilon, Zeta, Eta, Theta, Lota, Kappa, Lambda and Mu (classified as previous VOI) which will be easier and more practical to be discussed by non-scientific community [15]. According to GISAID, as of 16 May 2022 187 countries shared 3,648,731 Omicron genome sequences and 203 countries shared 4,425,174 Delta genome sequences [https://www.gisaid.org/hcov19-variants/] (accessed on 16 May 2022). 

The increase in the spread of VOCs has affected the efficacy of currently available vaccines. The phase III clinical trial results of the Oxford–AstraZeneca (ChAdOx1), Johnson & Johnson (Ad26.COV2.S), and Novartis (NVX-CoV2373) vaccines in South Africa have shown that the Vaccine Efficacy (VE) against the local Beta variant is decreased due to VOC [17,18,19]. For example, the protective efficacy of the Oxford–AstraZeneca (ChAdOx1) vaccine against Beta variant (mild to moderate illness) is only 10% [17]. Data from a gulf country showed that the Pfizer/BioNTech (BNT162b2) vaccine confers 87% and 72% protection against Alpha and Beta variants, respectively [20]. In Israel, with the emergence of the Delta variant, the overall efficacy of vaccines against infection has reduced from 95% to 39% [21]. In South Africa, the effectiveness of the BNT162b2 vaccine (two doses) against hospitalization for COVID-19 infection was significantly reduced from 93% in the non-Omicron period to 70% in Omicron period [22]. This decrease in the effectiveness of vaccines against the emerging VOCs has not only raised the concerns on the efficiency of vaccines but also pointed the likelihood of reinfection. Even though approved COVID-19 vaccines are less effective against the current circulating VOCs, on a brighter side, they remain to be highly effective in averting severe illness and death [23]. This review provides an update on the structure, genomic organization, and life cycle of SARS-CoV-2, VOCs (current and previous) and VOIs (previous). In addition, this review describes mutations reported in different variants and summarizes the most recent vaccines and their efficacies reported against emerging variants.

## 2. Structure and Genome Organization of SARS-CoV-2

SARS-CoV-2 virus was first reported [24] on 31 December 2019 [25]. Taxonomically, SARS-CoV-2 belongs to the realm Riboviria, order Nidovirales, suborder Cornidovirineae, family Coronaviridae, subfamily Orthocoronavirinae, genus Betacoronavirus (lineage B), subgenus Sarbecovirus, and the species severe acute respiratory syndrome-related coronavirus [26]. So far, there are seven different types of coronaviruses reported. Among these, four common human coronaviruses-229E, NL63, OC43, and HKU1—cause mild infections [27]. However, individuals infected with either of the other three coronaviruses—severe acute respiratory syndrome coronavirus (SARS-CoV), Middle East Respiratory Syndrome Coronavirus (MERSCoV), and SARS-CoV-2—develop severe respiratory distress and viral pneumonia and may ultimately succumb to the disease [28,29,30]. Jiang, Xiaowei, and Ruoqi Wang stated that although bats are the most probable reservoir animal for SARS-CoV-2 [31], zoonotic spillovers likely involving an intermediate animal and multiple transmissions from wildlife at a market in Wuhan probably led to SARS-CoV-2 emergence [32].

**Figure 1:**
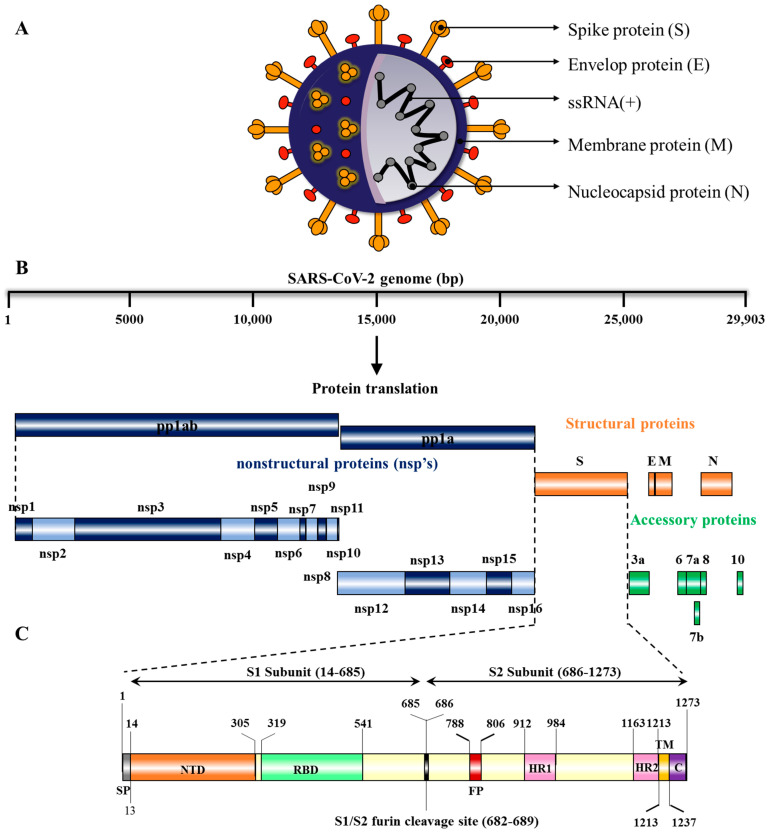
Genomic Organization and Structural Features of surface glycoprotein of SARS-CoV-2: (**A**) *Structure of SARS-CoV-2*: The schematic depicts the structural features of SARS-CoV-2 virus particle, which contains a single-stranded RNA, S-glycoprotein, and other structural proteins that include envelope (E), membrane (M), and nucleocapsid (N) proteins. (**B**) *Schematic representation of SARS-CoV-2*. *proteins:* Genome of SARS-CoV-2 consists of approximately 29,903 nucleotides, with ORF-1a and ORF-1b, which are translated to polyprotein 1a (pp1a) and 1ab (pp1ab), respectively. Four genes encoding for structural proteins such as spike (S), envelop (E), membrane (M), and nucleocapsid (N). Accessory proteins (ORF3a, 6, 7a, 7b, 8, and 10) are distributed among structural proteins. (**C**) *Structure of SARS-CoV-2 Surface glycoprotein (S):* The surface glycoprotein (S protein) is made up of 1273 amino acids, including N-terminal signal peptide (SP), S1 subunit, and S2 subunit. The S1 subunit contains an N-terminal domain (NTD) and a receptor binding domain (RBD), while the S2 subunit is composed of the fusion peptide (FP), heptapeptide repeat sequence 1 (HR1), HR2, TM domain, and cytoplasm domain (C).

SARS-CoV-2 (structure shown in Figure 1A) is a positive-sense, single stranded RNA virus whose genome size is ~29,903 bp and is organized in the following order from 5’ to 3’: open reading frame (ORF) 1ab (replicas), spike glycoprotein (S), ORF3a protein, envelope protein (E), membrane glycoprotein (M), ORF6 protein, ORF7a protein, ORF7b protein, ORF8 protein, nucleocapsid-phosphoprotein (N), and ORF10 protein (Figure 1B). The SARS-CoV-2 genomic RNA contains two major open reading frames (ORFs), ORF1ab and ORF1a, occupying 2/3rd of the genome (21,291 nucleotides or 1 to 21 kb) at the 5′ end and translated to polyprotein 1ab (pp1ab) and pp1a proteins. The virus genome encodes two proteases, a papain-like protease (PLpro), or nsp3, and a 3C-like protease (3CLpro), or nsp5, which cleave pp1a and pp1b polypeptides into 16 nonstructural proteins: leader protein, nsp2, nsp3, nsp4, 3C-like proteinase, nsp6, nsp7, nsp8, nsp9, nsp10, RNA-dependent RNA polymerase (RdRp), helicase, 3’–5’ exonuclease, endoRNAse, 2’-o-ribose methyltransferase, and nsp11 [32,33]. The remaining one third of the genome (21 to 29 kb) at the 3′-end has overlapping ORFs, encoding for at least four structural proteins: spike glycoprotein (S), envelope protein (E), membrane glycoprotein (M), and nucleocapsid protein (N) and six accessory proteins (ORF3a, ORF6, ORF7a, ORF7b, ORF8, ORF10) [33,34,35]. The genes and proteins expressed by SARS-CoV-2 along with their nucleotide location and number of amino acids are shown in Table 1.

**Figure 2:**
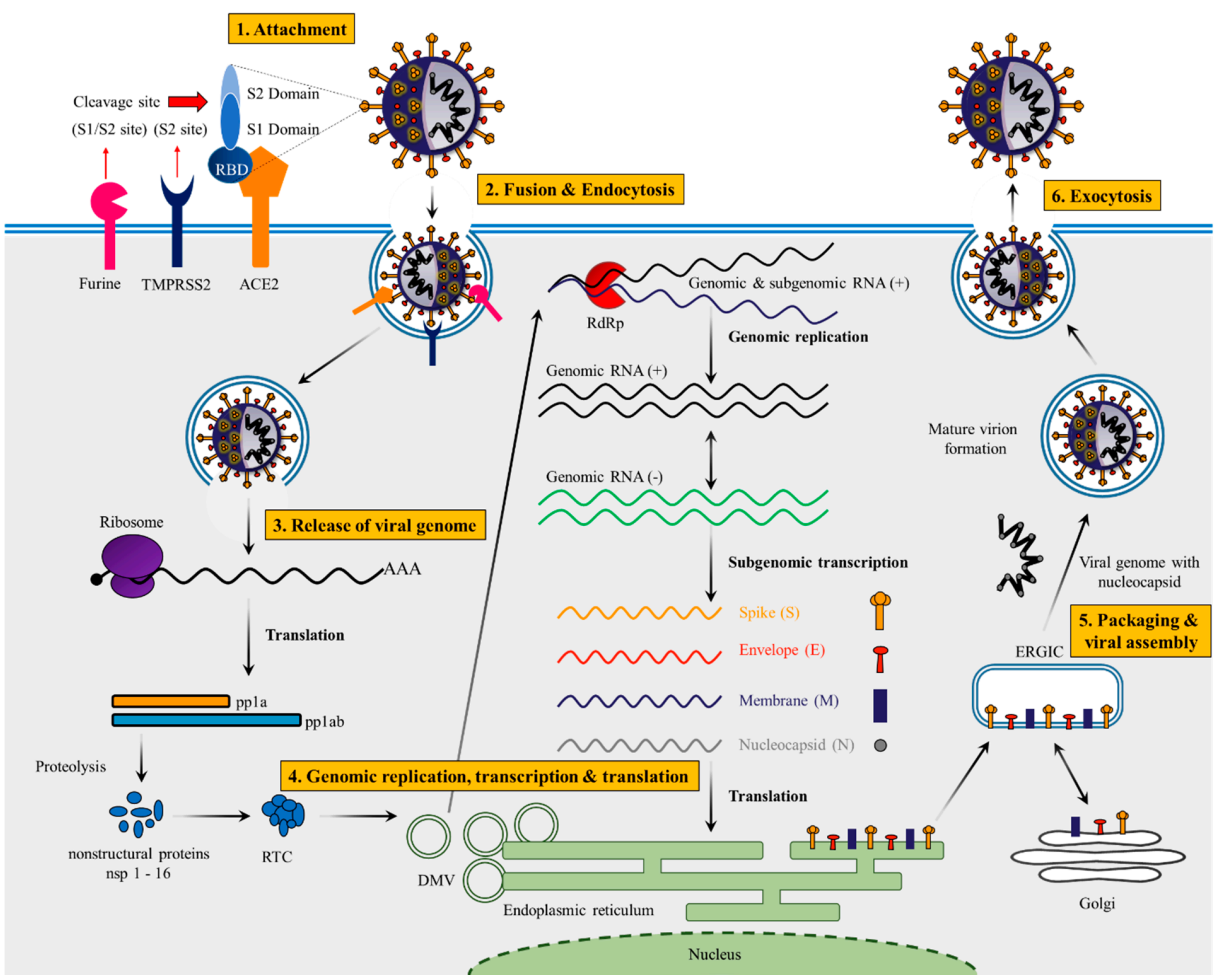
Complete life cycle of SARS-CoV-2. SARS-CoV-2 lifecycle begins with primary binding of the Spike protein to its specific receptors (ACE2). Mostly host cell entry depends on several steps: (i) Initial cleavage of S1/S2 specific sites by surface transmembrane protease serine-2 (TMPRSS2) and furine, (ii) followed by virus–cell membrane fusion and endocytosis. (iii) Continuing from endocytosis, m-RNA genome is mainly released into the cytosol and translated into the polyproteins. Mostly polyproteins (pp1a and pp1ab) are cleaved by specific viral-encoded protease (VEP) into the several nonstructural proteins (nsps) (mostly including RNA-dependent RNA polymerase: RdRp) which is responsible for replication transcription complex (RTC) in the cells. (iv) Viral replication begins with virus-induced double membrane vesicles mainly derived from the endoplasmic reticulum (ER). (v) Positive-strand of genome serves as a main template for full-length negative-strand RNA and sub genomic (sg)RNA and sgRNA translation results in both structural proteins and accessory proteins are inserted into the ER–Golgi intermediate compartment (ERGIC) for virion assembly, respectively. (vi) RNA genomes with specific nucleocapsid proteins are incorporated into newly synthesized virions, which are secreted by exocytosis. DMV—double-membrane vesicle.

The surface glycoprotein (S protein) is composed of 1273 amino acids, including the N-terminal signal peptide (SP) of 1–13 residues, the S1 subunit (14–685 residues) and S2 subunit (686–1273 residues) (Figure 1C). The S1 subunit is made up of an N-terminal domain (NTD, 14–305 residues) and a receptor binding domain (319–541 residues), while the S2 subunit is made up of the fusion peptide (FP, 788–806 residues), heptapeptide repeat sequence 1 (HR1, 912–984 residues), HR2 (1163–1213 residues), TM domain (1213–1237 residues) and cytoplasm domain (1237–1273 residues) [36]. Both S1 and S2 subunits are crucial in assembly and surface projection of the S protein, which interacts with Angiotensin-Converting Enzyme 2 (ACE2) receptors which are expressed on the lower respiratory pneumocytes of the host cell [30,33]. Host transmembrane Serine Protease 2 (TMPRSS2) cleaves the S protein at the furin cleavage site (682–689 residues) into S1 and S2 subunits, enabling viral fusion and entry [37,38] (Figure 2). After entering into the host cell, SARS-CoV-2 takeovers the host cell machinery to rapidly synthesize viral proteins, assemble, and release virus progenies [39,40].

## 3. Lifecycle of SARS-CoV-2

***The Infection Phase:*** SARS-CoV-2 lifecycle begins with the binding of the spike protein with ACE2 receptors. The spike protein consists of two subunits (S1 and S2). S1 subunit consists of receptor binding domain (RBD), which binds to the ACE2 receptors and enables viral attachment to cells [41,42,43,44,45], while the S2 subunit mediates viral cell membrane fusion by forming a six-helical bundle via the two-heptad repeat domain [46,47,48]. SARS-CoV-2 mainly utilizes the serine protease (TMPRSS2) for its entry into the host [49,50]. SARS-CoV-2 has a unique Furin cleavage site, which is required for the virus to enter cells that are lacking cathepsin protease [51]. Furin cleaves the SARS-CoV-2 spike protein at the S1/S2 site [52], thereby produce active S1 and S2 subunits. This fusion process occurs between the viral particles and hosts cell membrane, and play a crucial role in the viral infections.

***The ReplicationStage*:** After the release of viral genome into the host cell cytoplasm, replication begins with mRNA translation. For replication, viruses heavily depend on the host cell’s translation system to form its proteins [53]. The translation of two large ORF’s results in the synthesis of two large polyproteins (pp1-a and pp1ab), which takes place by ribosomal frameshifting of ORF1a and ORF1b [54]. The polyproteins pp1-a and pp1-ab formed during the process of mRNA translation and which are processed into 16 non-structural proteins (NSPs) and play an important role in the viral genome replication, transcription of subgenomic mRNA (sgmRNA) [55]. The functions, nucleotide location, and number of amino acids present in different NSPs are shown in Table 2. NSP-1 is mainly known for proteolytic release [56], which is also having role in the translation machinery in the host cells [57,58,59]. The remaining 15 NSPs (NSP2–16) consist of viral replication transcription complexes (RTC) which mainly accommodate the RTC; this facilitates the modulation of intracellular membranes, activation of co-factors, RNA synthesis, specific RNA modification, and finally host immune evasion [60,61,62].

**Maturation and release:** Coronavirus infection mainly accelerate the generation of endoplasmic reticulum (ER) derived perinuclear double membrane structures, double membrane vesicles, etc., which play a key role in viral life-cycle progression [63,64,65,66,67]. Initiation of viral replication followed by RNA synthesis, the m-RNA and specific N-proteins activate the translation and form the viral nucleocapsid. The S, E, and M proteins of m-RNA are inserted into the endoplasmic reticulum followed by translation by ribosomes [27,68,69]. During endoplasmic reticulum insertion, in translation process forming new secretory proteins and developing the endoplasmic reticulum–Golgi intermediate compartment (ERGIC) [70,71]. Here, several viral genomes are mainly encapsidated with several nucleocapsid proteins and activation of ERGIC membranes, budding off other proteins and developed as matured virions in the host cells [72]. Matured virions are separated and transported to the cell surfaces within the vesicles and finally released thorough exocytosis. Complete life cycle of SARS-CoV-2 is showed in Figure 2.

## 4. Variants of SARS-CoV-2

A specific group of viruses that inherit a characteristic mutation is called as a variant. Over the course of pandemic, numerous mutations and variants of SARS-CoV-2 have surfaced in the world. From the evolutionary perception, variants that attain competitive benefit with respect to viral replication, transcription, and escape from the host immunity are presumably to survive and increase their frequency. RNA viruses such as SARS-CoV-2 mutate more gradually than most RNA viruses due to proofreading mechanism during replication, which results in less mutations and higher precision in virus replication [73]. SARS-CoV-2 variants were first reported in early March 2020 having a single D614G mutation in the spike (S) glycoprotein, and variants having this particular mutation preponderated since June of 2020 [7], possibly due to boosted viral fitness and transmissibility [74,75]. Although few vaccines, like BNT162b2 and mRNA-1273 have shown promising outcomes, with over 95% protective effectiveness against COVID-19 [76,77], these interventions were directed only towards the preliminary SARS-CoV-2 virus that emerged in 2019. The current development of new SARS-CoV-2 variants is a matter of concern due to several mutations in the spike protein. Such mutations could influence the structure of the protein, thereby varying infection rates by amending the interaction of the spike protein with the human hACE2 receptor, modifying immune response, or compromising the effectiveness of treatments by MAB’s (monoclonal antibodies). With the continued emergence of multiple variants, U.S. Department of Health and Human Services established a SARS-CoV-2 Interagency Group (SIG), which focuses on the rapid characterization of emerging variants by evaluate the risk posed by SARS-CoV-2 variants circulating in the United States and make recommendations about the classification of variants [78]. Taking into consideration the continuous evolution of SARS-CoV-2 variants and their impact on public health, variants may be reclassified based on their attributes and prevalence in the United States. SARS-CoV-2 Interagency Group (SIG) classified variants into four major categories: they are VBM, VOC, VOI, and VOHC [78]. Alpha (B.1.1.7), Beta (B.1.351), Gamma (P.1), Delta (B.1.617.2), Epsilon (B.1.427, B.1.429), Eta (B.1.525), lota (B.1.526), Kappa (B.1.617.1), Mu (B.1.621), and Zeta (P.2) are classified under VBM. Omicron (B.1.1.529) is classified under VOC. Currently, there are no variants classified under VOI and VOHC categories [78].

### 4.1. SARS-CoV-2 Variant of Concern

**Figure 3:**
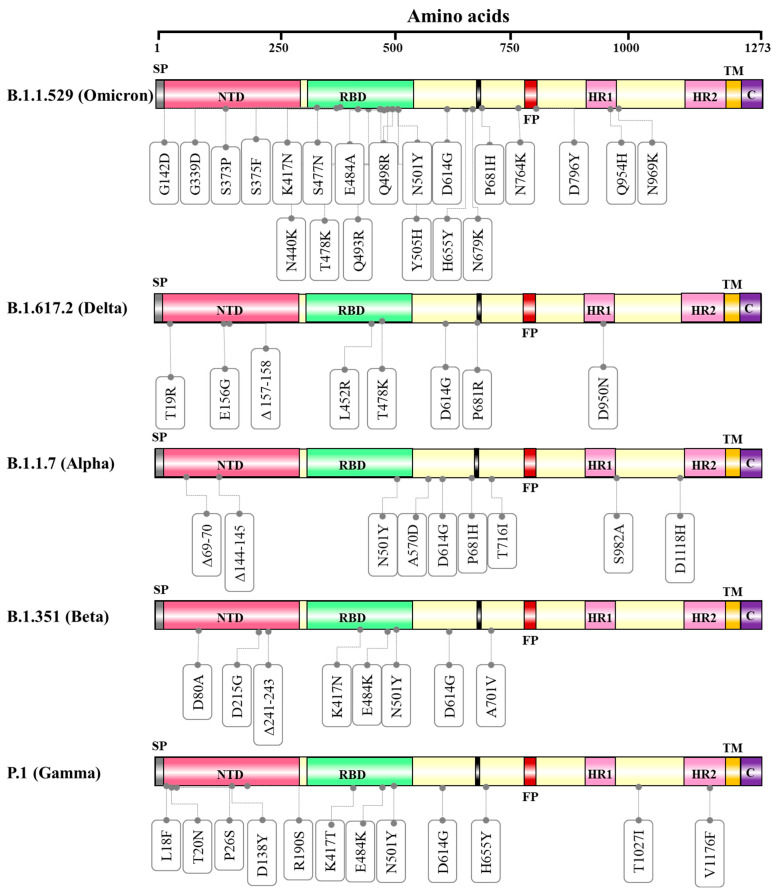
Mutations reported in amino acid positions of spike glycoproteins (S) in current circulating variant of concern (VOC): Omicron (5.1.1.529) and some Variants Being Monitored: Delta (B.1.617.2), Alpha (B.1.1.7), Beta (B.1.351), and Gamma (P.1).

As stated by CDC, VOC can be defined as “variant for which there is evidence of an increase in transmissibility, more severe disease (for example, increased hospitalizations or deaths), significant reduction in neutralization by antibodies generated during previous infection or vaccination, reduced effectiveness of treatments or vaccines, or diagnostic detection failures” [78]. According to CDC, as of 22 August 2022, the current circulating Variant of Concern (VOC) in the United States is only Omicron (B.1.1.529) [78]. Current circulating Variant of Concern, other names, and mutations reported are shown in Table 3. Mutations reported in amino acid positions of spike glycoproteins (S) are shown in Figure 3.

#### The B.1.1.529 (Omicron Variant)

Until November 2021, the Delta variant was designated as VOC. According to WHO, on 24 November 2021, a new lineage of SARS-CoV-2 was reported to the WHO by South Africa for the first time, and on 26 November 2021, the WHO’s Technical Advisory Group on SARS-CoV-2 Virus Evolution stated Pango lineage B.1.1.529 as VOC and designated it with the Greek letter omicron [79,80]. According to GISAID as of 16 May 2022, 3,648,731 Omicron genome sequences have been shared by 187 countries and is responsible for triggering the ongoing fourth wave of the COVID-19 epidemic [80,81]. The Omicron variant is the most heavily mutated variant among all the VOCs so far, which paves the way for enhanced transmissibility and partial resistance to immunity which is enhanced by COVID-19 vaccines [82]. The threat induced by Omicron is closely related to its effect on current vaccine efficacy because a total of 87% of these Omicron-infected individuals were completely vaccinated, boosted, or formerly infected with SARS-CoV-2 [83]. 

Genome sequenced data of the Omicron variant confirmed more than 30 mutations in the spike protein by which the SARS-CoV-2 protein recognizes host cells [84]. The more worrying are the 15 mutated sites (G339D, S371 L, S373P, S375F, K417N, N440K, G446S, S477N, T478K, E484A, Q493K, G496S, Q498 R, N501Y, and Y505 H) in the receptor-binding domain (RBD) that interacts with human cells before cell entry, covering nearly all of the significant mutations in the preceding VOCs (Alpha, Beta, Gamma, and Delta) [85,86,87]. Analysis of these mutations indicates the chance of increased transmission by evading the immune response [85] and have a huge impact on the effectiveness of vaccines and monoclonal antibody drugs [88,89]. The N501Y mutation enhances the binding affinity of the SARS-CoV-2 virus with the ACE2 receptor, which is a major influencer of increased transmission, and in combination with Q498R, the binding affinity gets stronger, and the Omicron variant gets easy access into the host [85]. Moreover, the risk of reinfection of previously infected COVID-19 patients with the Omicron variant is very evident, indicating higher transmissibility [90]. Omicron variant mutations H655Y and N679K are present near the furin cleavage site (FCS), can increase spike cleavage, making the virus more contagious [91,92] and on the other hand, P681H can multiply transmissibility by increasing the spike protein cleavage [93]. Furthermore, the Omicron variant gives a false negative result in polymerase chain reaction tests because of the “S gene target failure,” which paves the way of spreading the infection at a higher speed worldwide [82]. The Characteristic mutations (in at least 75% of sequences) in B.1.617.2 lineage are **ORF1a**—T3255I, P3395H, P314L, I1566V **S**—G142D, G339D, S373P, S375F, K417N, N440K, S477N, T478K, E484A, Q493R, Q498R, N501Y, Y505H, D614G, H655Y, N679K, P681H, N764K, D796Y, Q954H, N969K **E**—T9I **M**—Q19E, A63T **ORF8**—S84L **N**—P13L, del131/133, R203K, G204R [94]. 

Sublineages of the Omicron variant: Genomic sequence and computational analyses have divided the Omicron variant (B.1.1.529.1 or BA.1) into several sub-lineages, BA.1.1 (B.1.1.529.1.1), BA.2 (B.1.1.529.2), and BA.3 (B.1.1.529.3), BA.4 (B.1.1.529.4), and BA.5 (B.1.1.529.5) [95,96]. In the evolutionary descent, BA.1.1 sublineage developed first, followed by the BA.2 and BA.3 lineages [97]. These three sub-lineages BA.1.1, BA.2, and BA.3, are closely related to a common ancestor [98,99]. The BA.1.1 sub-lineage is with a unique substitution in the S-protein, i.e., R346K [15]. The BA.2 sub-lineage is distinguished from BA.1 by the presence of eight changes in its S-protein [100]. In comparison to BA.1 sub-lineage, BA.2 sub-lineage has three new mutations, viz., T376A, D405N, and R408S, but lacks the G446S and G496S, which are seen in BA.1 [101]. The BA.2 sub-lineage, which produced the winter spike of coronavirus disease 2019 (COVID-19) instances in January 2022, appears to be more infectious than the Omicron BA.1 sub-lineage. When compared to the original Omicron strain, the BA.2 sub-lineage is reported to be more significantly immunologically resistant and has stronger cell fusion than BA.1 sub-lineage [97,102]. By analyzing the replication process in human nasal epithelial cells, researchers observed that the virological properties of the BA.2 sub-lineage were more infectious than the BA.1 lineage [89,98,103]. In several nations, the BA.1 has been superseded by the BA.2 sublineage [101,104]. Furthermore, BA.1 and BA.2 sub-lineages have distinct variances in their susceptibility to therapeutic monoclonal antibodies [104]. In South Africa, researchers have discovered evidence of the existence of two additional sublineages, named BA.4 and BA.5. According to recent research, the newly discovered sublineages have similar RBD sequences to BA.2, but with the addition of L452 and F486 substitutions. BA.2.12.1 (L452Q), BA.2.13 (L452M), BA.4, and BA.5 (L452R+F486V) have a larger transmission advantage over BA.2. It is plausible that the formation of these sublineages in this region is related to much lower immunization rate when compared to other nations [105]. These developing sublineages are spreading faster than other previously circulating strains, most notably BA.2, which prompted a rise in infections at the start of the year. However, the most recent Omicron subvariants appear to be causing fewer fatalities and hospitalizations than their older counterparts, indicating that rising population immunity is dampening the immediate repercussions of COVID-19 outbreaks [106]. Furthermore, new evidence reveals that the Omicron is constantly developing in response to immunological pressure, which explains the occurrence of R346K (BA.1.1), L452 substitutions, and F486V mutations, all of which facilitated better immune evasion. Unlike original Omicron, the Omicron sublineages now have the potential to target humoral immunity. The Omicron breakthrough infections mostly recall wild type (WT)-induced memory B cells [107,108], narrowing the range of antibodies evoked and perhaps facilitating the development of subsequent mutants. In vitro tests of sera from unvaccinated individuals who have previously been infected with BA.1 show that both BA.4 and BA.5 are capable of evading the protection provided by BA.1 [109,110]. These incidences provide a significant challenge to the herd immunity that has been acquired by WT-based vaccination and BA.1/BA.2 infection. The enhanced proliferation and dominance of BA.4 and BA.5 have been linked to their ability to evade immunological defense acquired by the previous infection and/or vaccination, particularly if the humoral immune response has diminished over time. Additional investigations are immediately needed to elucidate the mechanisms of receptor binding and immune evasion abilities of the novel variants [101].

### 4.2. SARS-CoV-2 Variants Being Monitored

As stated by CDC, VBM can be defined as variants whose “data indicated there is a potential or clear impact on approved or authorized medical countermeasures or that have been associated with more severe disease or increased transmission but are no longer detected, or are circulating at very low levels, in the United States” [78]. These variants do not pose a significant risk to public health in the United States [78]. According to CDC, as of 22 August 2022, the current circulating VBM in the United States are Alpha (B.1.1.7), Beta (B.1.351), Gamma (P.1), Delta (B.1.617.2), Epsilon (B.1.427, B.1.429), Eta (B.1.525), lota (B.1.526), Kappa (B.1.617.1), Mu (B.1.621), and Zeta (P.2) [78]. List of current circulating Variants Being Monitored (VBM), other names, and mutations reported are shown in Table 4. Mutations reported in amino acid positions of spike glycoproteins (S) of some important Variants Being Monitored are shown in Figure 3. Among the listed VBM, only the Delta variant is discussed in the current review because of its rapid spread and being a Variant of Concern till the second week of April 2022.

#### B.1.617.2 Lineage (Delta Variant)

The Delta variant was first discovered in India in October 2020, and began to spread rapidly in early April 2021, resulting in a third wave of outbreaks around the world [111,112]. This particular variant has three sub-lineages: B.1.617.1, B.1.617.2, and B.1.617.3. Along with the primary D614G mutation, there are a couple of specific mutations for the Delta variant in the spike protein, one at position 452 (RBD region) and another at position 681 (furin cleavage site between S1 and S2). The Delta variant is an exception in the B.1.617 lineage as it does not have replacement at position 484 [113]. On 10 May 2021, the WHO designated B.1.617 and its sub-lineages, namely B.1.617.1 (Kappa), B.1.617.2 (Delta), and B.1.617.3, as ‘VOC’ after Alpha, Beta, and Gamma [10]. On 1 June 2021, WHO stated that only the B.1.617.2 sub-lineage (designated the Delta variant), remained as VOC, and sub-lineage B.1.617.1 (designated Kappa variant) was classified as a VOI. Coronaviruses from this lineage (B.1.617) has shown increased transmissibility, secondary attack rate, and also showed similar transmissibility between vaccinated and unvaccinated individuals, resulting in severity of the disease [114]. According to GISAID as of 16 May 2022, more than four hundred thousand sequences in the Delta lineage have been reported since the lineage was identified, and the strain has been reported in 203 countries. The Characteristic mutations (in at least 75% of sequences) in B.1.617.2 lineage are ORF1a—A1306S, P2046L, P2287S, V2930L, T3255I, T3646A ORF1b—P314L, G662S, P1000L, A1918V S—T19R, E156G, del157/158, L452R, T478K, D614G, P681R, D950N ORF3a—S26L M—I82T ORF7a—V82A, T120I ORF7b—T40I ORF8—S84L, del119/120 N—D63G, R203M, G215C, D377Y [94]. The B.1.617 lineage harbors several mutations in the S protein including T19R, G142D, E156G, del 157/158, L452R, T478K, D614G, P681R, and D950N. Studies suggest that the Delta variant (B.1.617.2) is 40 to 60% more transmissible than the Alpha (B.1.1.7) variant and may be the most contagious variant the world has seen as of June 2021 [115].

The Delta variant is known for its shorter incubation period and higher virus load. For example, a study showed that the Delta variant viral load is one thousand times higher compared to the ancestral strain viral load [116]. According to the Public Health England (PHE) report, Delta variant attacks 51%−67% more than Alpha variant [117]. The increase in transmissibility of the B.1.617.2 variant is mainly because of the mutation in the furin cleavage site (P681R), which increases viral entry into host cells; however, this particular variant lacks mutations in the ACE2 receptor binding domain (amino acid positions 501 or 484), commonly associated with VOCs or escape from neutralizing antibodies (NAbs) [118,119]. Another critical mutation which helps in allowing this variant for transmission from one individual to another individual is in the RBD (L452R). This particular L452R mutation is anticipated to increase the transmissibility by 18%–24%, reduction in the neutralization titers in the vaccinated individual by 20-fold, and also resistance to neutralization by specific antibodies [120].

A novel variant of Delta known as ‘Delta plus’ was first detected by PHE on 11 June 2021. Apart from mutations that are common with Delta variant it has an additional mutation (K417N) which might contribute to immune escape. Based on the analysis of 38,805 sequenced cases in England, PHE reported that when compared to Alpha variant Delta variant was associated with >2 times higher risk of hospitalization within 14 days of specimen date. A total of 73% of Delta cases are reported in unvaccinated people, and only 3.7% Delta cases are reported in people who are completely vaccinated, indicating that the Delta variant is likely to spread rapidly among unvaccinated groups of individuals. Reduced serum antibody neutralization against Delta variants has been reported [121], and a pseudovirus with the spike of the Delta variant reduced mRNA-vaccinated serum neutralization by almost 2.9-fold [122]. According to a live virus experiment, The Kappa lineage (B.1.617.1) is 6.8-fold more resistant to neutralization by sera from COVID-19 convalescent patients and Moderna and Pfizer vaccinated individuals [123].

## 5. Vaccine Candidates Approved against SARS-CoV-2 Infection

According to WHO, as of 13 May 2022, a total of 354 COVID-19 vaccine candidates are in various stages of development, with 156 in clinical and 198 in preclinical development. These vaccine candidates were developed based on a variety of approaches, including Protein subunit (PS), Viral Vector (non-replicating) (VVnr), DNA, Inactivated Virus (IV), RNA, Viral Vector (replicating) (VVr), Virus Like Particle (VLP), VVr + Antigen Presenting Cell (VVr + APC), Live Attenuated Virus (LAV), VVnr + Antigen Presenting Cell (VVnr + APC), and Bacterial antigen-spore expression vector (BacAg-SpV). Table 5 illustrates the distribution of various COVID-19 vaccine candidates among different platforms. Out of 156 vaccines in clinical development, protein subunit vaccines (N = 52) constitute the largest category, accounting for 34% of all the COVID-19 vaccine candidates being developed. As of 13 May 2022, there are 38 vaccines of different categories which have been authorized in at least one country for combating the SARS CoV-2 pandemic [https://covid19.trackvaccines.org/] (accessed on 13 May 2022). A list of vaccines authorized for combating the SARS CoV-2 and their reported efficacies against different variants is shown in Table 6.

### 5.1. Messenger RNA (mRNA) Vaccines

Although mRNA vaccines are characterized by triggering the robust immune responses in the host cells, they act as intrinsic adjuvants and show favorable safety profiles in individuals. These vaccines are low-cost preparations or formulations, contain suitable storage conditions, and are easy to administer [1]. For the first time, the mRNA vaccine preparation technology was used for human vaccination in treating SARS-CoV-2. Previously, mRNA vaccine preparations were used for different viruses such as Rabies and Zika [124]. Mechanistically, mRNA vaccines allow the body cells to produce specific S-protein rather than injecting them into the host cells; however, it reduces the time required for building the vaccine efficiency, hence these vaccine platforms require less time compared to the other conventional vaccines [125]. The mRNA vaccines use targeted nucleosides, which contain single strand mRNA for efficient delivery of genetic materials. In the formulation, the encapsulated lipids protect mRNA from the degradation by host cells. After the administration of mRNA vaccines, the S-protein is produced within the cells and subsequently, the immune responses are initiated with the release of specific cytokines such as humoral antibodies and T-cell specific antibodies (CD4+ and CD8+) and the antibodies neutralizing the S-protein and its fragments [126].

#### 5.1.1. Pfizer–BioNTech Vaccine (Comirnaty (Formerly BNT162b2))

Comirnaty (BNT162b2) (generic name-tozinameran) is a nucleoside modified mRNA-based vaccine, manufactured by the Pfizer and BioNTech. PBV vaccines are given in a two-dose regimen within the 3-week time interval. Individuals are recommended to receive a booster shot or third dose within 12 months. PBV vaccines are mainly supplied in the form of liquid for 5 shots (two extra shots). According to earlier reports, PBV vaccine efficacy showed a strong protection against SARS-CoV-2 within the 10–14 days after administration of first dose and irrespective to the race, and weight [127]. PBV requires ultra-cold temperatures (−70 °C) for storage and distribution, which imposes difficulties for distributing this vaccine into remote and rural areas [76]. 

Clinical studies with Comirnaty have reported an about 95% efficiency with a gradual decline in a period of 6 months [76]. Comirnaty showed similar immunogenicity profile as BNT162, which was devolved by the Pfizer [128]. Additional studies have shown that Comirnaty is relatively less effective against the SARS-CoV-2 virus as the virus has a modified spike protein mutation similar to the B.1.351(Beta) variant [129]. Two doses of the vaccine exhibited 88% effectiveness against B.1.617.2(DELTA) variant [123]. Recent reports have shown the efficacy of Comirnaty even against Omicron variant [22].

#### 5.1.2. Moderna Vaccine (MV) (mRNA-1273)

The Moderna Vaccine (MV) is a two-dose vaccine manufactured by Massachusetts based Pharmaceutical Company in collaboration with the National Institute of Health (NIH). MV is a mRNA-based vaccine, wherein mRNA-1273 is encapsulated in lipid Nano particles. It is also called as TAK-919 in Japan. MV has many advantages over the PBV. MV can be stored at −20 °C, and hence is relatively easier to transport. According to pre-clinical studies, MV has minimal side effects and elicits a stronger immune response in children and adolescents compared to cancer patients and pregnant women.

On 30 April 2021, WHO issued an emergency use listing (EUL) for MV. In the phase 3 trial, MV showed an efficacy of 94.1% (COVE trial; NCT04470427) [130]. Similarly, in a phase 2/3 trial conducted in 3225 individuals aged between 12 and 18 years, MV showed 96% efficacy in individuals who had received one injection (NCT04649151). Trials testing the allergic responses to MV are currently under progress (NCT04761822). Morbidity and Mortality Weekly Report (MMWR) of CDC, which highlights the latest scientific information pertaining to the safety and efficacy of COVID-19 vaccines, showed a 92% efficacy for MV. In addition, MMWR highlighted that MV is 95% effective in preventing hospitalization of COVID-19 individuals [131]. MV exhibited protective effects against B.1.351(BETA) and B.1.429 (EPSILON) variants; however, was less effective against B.1.315. Based on the clinical data, the vaccine is effective (85% efficacy) against the Omicron variant [132]. However, the efficacy levels dropped to ~55% after 7 months of administration [133].

### 5.2. Human Adenovirus Non-Replicating and Replicating Vector-Based Vaccines

Adenoviruses (Ads) are non-enveloped, icosahedral, double-stranded DNA viruses that can infect vertebrates including humans and non-human primates. Viral vector vaccines utilize the attenuated replicon as a component or replicon deficient components as viral backbones for transformation [134,135]. Adenoviruses are the most common viral vectors used for the efficient delivery of a plasmid containing a fragment of DNA coding for SARS-CoV-2 S-protein. After administration, viral vector-vaccines enter the host cells and deliver the genetic material into the nucleus. Once transcribed, the mRNA leaves the nucleus and is translated into the spike proteins and their tiny fragments. These tiny fragments are recognized by the host immune system and generate the specific neutralizing antibodies, CD4+ and CD8+ T-cells and B-cells and memory T-cells [136,137]. Viral vector vaccines contain the protein coat, which helps in the protection of the genetic materials, hence, no frozen conditions are required during transportation, and can be stored for a minimum of 6 months at 2 °C to 8 °C. According to recent investigations, viral vector vaccines induce a strong immune reaction, hence, adjuvants are not required for eliciting a strong immune response.

#### 5.2.1. Oxford–AstraZeneca Vaccine (AZD 1222)

The AZD 1222 vaccine is produced by Oxford University in collaboration with AstraZeneca. AZD 1222 was developed using vector based recombinant chad0x1 in chimpanzees. AZD 1222 vaccine was approved for usage for individuals aged over 18 years at 2 interval doses. This vaccine is proven safe and demonstrated to exhibit maximum efficacy in combating the SARS-CoV-2 [138]. The two-dose vaccine reported an efficacy, which ranges from 62% to 90%. The strong humoral and cellular immune responses triggered in host cells by the vaccine are critical in determining the efficacy of vaccine. AZD 1222 can be stored in refrigerator at temperature 2 °C to 8 °C for at least 6 months. The vaccine administration showed a few minor side effects such as fatigue and headache [139]. A phase-2 testing was also conducted using AZD 1222 (NCT04283461). Based on the recent reports by CDC’s MMWR, the vaccine exhibited not only an efficacy of 92% in preventing SARS-CoV-2 but reduced the hospitalization in 95% cases [123]. A phase-3 study reported an overall estimated vaccine efficacy of 74.0% and estimated vaccine efficacy was in participants with 65 years of age or older 83.5% [140]. Vaccine efficacy against omicron variant showed that Spikevax is 85% effective for 30 days after the administration, but the efficacy has declined to 55% after 7 months [141].

#### 5.2.2. Sputnik-V Vaccine (SVV)

Sputnik-V vaccine also called as SVV, was named in the memory of the Soviet era. The vaccine is based on vector technology and it was produced by the state research Centre of Viral Biotechnology and Gamaleya Institute. It is administered in two doses 21 days apart. This vaccine utilizes a combination of two adenoviruses such as ad-5 and ad-26, which are primarily recognized by the host cells of the human immune system [142]. Intra muscular SVV exhibited an efficacy of 91.6% [143]. SVV utilizes ad-26 vector for the first dose and ad-5 vector for the second dose of administration. The company also provides a one dose variation, which could produce short time immune response with a reported efficacy of 73–85%. The vaccine can be stored in the freezer at −20 °C, and recently Sputnik-V and AstraZeneca together tested the combination of both vaccines for combating SARS-CoV-2. This combination is effective (91.6% efficacy) when tested in a phase-3 clinical trials consisting of 21,997 individuals [143]. Several reports also postulated that the vaccine has good safety profile and stimulates strong humoral and cellular responses in COVID-19 patients [144]. According to the CDC’s MMWR results vaccine has 92% efficacy in preventing the COVID-19 individuals in emergency condition and was 95% effective in preventing the hospitalization [145]. SVV showed 90% efficacy against B.1.617.2 (DELTA) variant. According to recent data, SVV maintains sera that neutralizes the B.1.1.7 (ALPHA), B.1.315 (BETA), P.1 (GAMMA), and the two versions of B.1.617(DELTA) variants [146]. 

#### 5.2.3. AD5-nCoV Vaccine (PakVac, Ad5-nCoV)

AD5-nCoV, also called as CONVIDECIA, is manufactured by Can-Sino Biologics Company, China in collaboration with the Academy of Military Medical Sciences, China. This vaccine utilizes ad-5 adenovirus [147]. Presently this vaccine is in phase-3 clinical trial; but the Chinese government has approved this vaccine for use in military individuals for a specific period of one year. According to recent reports, the vaccine efficacy is 65.7% for a single dose of intramuscular administration [147]. The vaccine can be stored at 2 °C to 8 °C. It had no severe adverse effects. A phase 1 trial stated that administration of this vaccine produced humoral and immunogenic responses in the target site. A phase-3 trial showed that a single short of Convidicea is 57.5% effective against symptomatic COVID-19 for at least 28 days [148]. 

#### 5.2.4. Ad26.COV2.S/Janssen (JNJ-78436735; Ad26.COV2.S)

This vaccine is manufactured by the Janssen Pharmaceutical Company. Recently, FDA granted approvals for this vaccine for emergency use as a single dose of administration [149]. The vaccine is a carrier/viral vector vaccine and has shown more potential in reducing the spread of the virus in vaccinated individuals. Common side effects of this vaccine include fatigue, fever, headache, and myalgia. The developed vaccines (two-dose versions) were tested in a double blinded, randomized, placebo controlled phase1/2 study (NCT04436276) and also in a phase 3 trial (JNJ-78436735), formerly called as ensemble (NCT04614948). Currently, NIH-funded trial is evaluating the safety and immunogenicity of a specific booster dose of Moderna vaccine for individuals who had received one dose of Janssan vaccine (NCT04889209). Based on the clinical data, vaccine efficacy was reported to be 52.9% against moderate or critically effected COVID 19 individuals [150]. A phase 1/2 study in humans reported that single dose of vaccine showed a significant specific immunogenicity and a good safety profile [151]. The vaccine had an efficacy of 66.9% at 14 days of administration and 66.1% at 28 days of administration [19]. J&J announced that the vaccine elicited neutralizing antibodies against the B.1.617.2 (DELTA). 

### 5.3. Inactivated Coronavirus Vaccines

Recently, it was reported that viral variants can multiply in monkey kidney cells and grow in the bioreactors. Four inactivated vaccines have been given authorization for use in humans, while some of the other inactivated vaccines are in phase I/II clinical trials or in preclinical development. Inactivated vaccines express a wide range of viral antigens, which can induce a TH-2 response and stimulate broad spectrum of immune responses [152].

#### 5.3.1. Sinopharm Vaccine (BBIBP-CorV)

The SV vaccine, also called as BBIBP-CorV, is manufactured by the Sinopharm which is a Chinese owned company and vaccines are marketed with the help of UAE. The vaccine utilizes the inactivated viral technology [153]. These vaccines are administrated in two-dose regimen through intramuscular route. The vaccine showed an efficacy of 79.34% in China and 86% in the UAE and did not report any adverse effects. Interestingly, the effectiveness of this vaccine in pregnant women showed comparable result to non-pregnant women of similar ages [153]. A phase 1 trial result showed that BBIBP-CoV vaccine is safe and well tolerated at the dosing level; however, participants showed humoral responses to the vaccine after the 42 days of administration [154]. A phase 3 BBlBP result testing the combination of BBIBP-CorV and WIBP-CoV showed that the BBIBP is more effective and produced about 78% efficacy in the group, which received aluminum hydroxide compared to the control group [155]. 

#### 5.3.2. SARS-CoV-2 Vaccine (Vero Cells)

This vaccine, also called as Vero vaccine, is manufactured in collaboration with Institute of Medical Biology and Chinese Academy of Medical Sciences [156]. This vaccine utilizes the inactivated viral technology [157]. These vaccines are given to the individuals in two-dose intervals intramuscularly [157]. Vaccine was found to be safe and immunogenic in a phase 1/2 trial consisting of 191 participants in the phase 1 portion of the phase 1/2 trial [158].

#### 5.3.3. Sinopharm-Wuhan Vaccine (WIBP-CorV)

Sinopharm vaccine is also called as SWV. It is manufactured by the Wuhan Institute of Biological Products company in China. The vaccine was effective in preventing the SARS-CoV-2 with 72.5% efficacy. However, it showed some side effects. It is currently in phase-III clinical trial [136]. These vaccines utilize the WIV-40 strain, which is mainly isolated and cultivated in a Vero-cell line for their propagation. The interim analysis of two randomized control trails data suggested that seroconversion rate is 100% in phase-I clinical trials and 85.7% in phase-II trails [136]. These vaccines are given to the individual in two dose intervals and in some cases, a third dose is also recommended for the individuals who show weak immune responses. Results from phase 1 and phase 2 trials showed that WIBP-CorV is strongly immunogenic. In May 2021, results of the phase 3 trial involving both BBIBP-CorV and WIBP-CorV showed that WIBP-CorV was 72.8% effective compared with the control group that received only aluminum hydroxide [155].

#### 5.3.4. CoviVac

The CoviVac vaccine was developed by the Federal Scientific Centre for Research and Development of Immune and Biological products. On 20 February 2021, the third domestic vaccine against COVID-19 was, officially approved for the domestic use in Russia. These vaccines are given to the individuals in two dose intervals intramuscularly [159]. A phase III trial involving this vaccine has been started and it is expected to end on 30 December 2021. According to the ministry of science and higher education of the Russian Federation (http://www.chumakovs.ru/en/ (accessed on 29 May 2022)) CoviVac was initially evaluated in 30 individuals aged between 18–45 and a phase 1/2 trial is currently being conducted in Russia in 300 individuals.

#### 5.3.5. CoronaVac Vaccine

Corona vaccine, formerly called as PiCoVacc, is manufactured by a Beijing based pharmaceutical company SinoVac Biotech in collaboration with Brazilian Research Centre, Butantan. This vaccine utilizes inactivated viral technology, and is administered in two doses intramuscularly, 14–28 days apart. These vaccines partially activate the immune response and produce low levels of antibody in SARS-CoV-2 individuals. Therefore, this vaccine requires adjuvants such as alum, to increase the immune responses while producing fewer side effects [136]. The vaccine was found to be effective even in the children. The vaccine can be stored and transported at 2–8 °C. The booster dose regimen of CoronaVac was also tested and compared with the Pfizer vaccine in randomized controlled trails (NCT04611243). Recently, a specific version of the CoronaVac, which specially target B.1.1.529 (Omicron) variant was developed [160]. CoronaVac showed 65.9% for the prevention of COVID-19 and 87.5% for the prevention of hospitalization, 90.3% for the prevention of ICU admission, and 86.3% for the prevention of COVID-19–related death [161]. Based on the preliminary results CoronaVac was found to be ineffective against the Brazil P.1 variant. Recent reports have shown that BBIBP-corV and CoronaVac were equally effective against the B.1.1.7 variant present in the UK, and less effective against B.1.351 variant present in South Africa [160].

#### 5.3.6. Inactivated SARS-CoV-2 Vaccine (Vero Cell)

This vaccine is manufactured by collaboration of Shenzhen Kangtai Biological Products/Beijing Minhai Biotechnology co-limited based in China. The phase 2 trials included 18 to 59 years as suitable age groups for testing this vaccine. In April 2021, the company registered a phase 3 trial, which was launched in May 2021. The Chinese government granted an emergency use approval for this vaccine in May 2021 [162].

#### 5.3.7. Covaxin (BBV152)

Covaxin, also called as a BBV152, was manufactured by the Bharat Biotech company in collaboration with the Indian Council of Medical Research (ICMR) and National Institute of Virology (NIV). The vaccine utilizes the inactivated viral technology, and the prescribed two doses are recommended to administer intramuscularly. In India, Covaxin has been approved for emergency use, despite being in phase-3 clinical trials [163,164]. Bharat Biotechnology Company also signed a partnership with Ocugen Pennsylvania, USA for marketing the Covaxin in USA. Covaxin can be stored at room temperature for a week [163].

A phase-I Covaxin clinical trial data showed tolerable safety outcomes and increased specific immune responses. A phase-III trial conducted in 25,800 participants was completed in January, 2021(NCT04641481). A phase 2/3 trial in pediatric participants (2–18 years of age) was recently completed (NCT04918797). Covaxin efficacy was reported to be 77.8% effective in preventing COVID-19 [165]. Additional reports testing the safety and immune responses of Covaxin showed no toxicity; and appeared to launch an effective immune response against SARS-CoV-2. Recent reports have demonstrated that Covaxin is effective even against B.1.1.7 and B.1.617 variants [166]. A case control study conducted in India reported that Covaxin is dominated by the immune evading delta variant (B.1.617.2). Recent data, which are in pre-print, showed that Covaxin could trigger a robust immune reaction against multiple variants for a minimum period of 6 months after administration [167]. Further studies should test Covaxin efficacy against newly emerging SARS-CoV-2 variants.

#### 5.3.8. QazVac (QazCovid-in)

Qazvac vaccine, also known as qazCovid, is developed by the Research Institute for Biological and Safety Problems at Kazakhstan [168]. A second vaccine called as QazCoVac-P, which is in clinical trials, was also developed by this institute. QazVac is recommended for individuals over 18 years with two doses administered across a 21-day period [169]. Between 1 September 2020 to 19 December 2020, the company has conducted a phase 2 trial and reported that the participants had a robust immune response against SARS-CoV-2 [NCT04530357]. The vaccine is stable between 2 °C to 6 °C. QazVaccine is currently recommended for the human use against SARS-CoV-2. A phase 3 trial testing the efficacy in 3000 individuals (NCT04691908) reported an efficacy of 96% (https://fortune.com/2021/04/26/new-covid-19-vaccine-kazakhstan-qazvac/ (accessed on 21 June 2022).

#### 5.3.9. COVIran

CoVIran vaccine also called as Barekat, is manufactured by the Shifa Pharm Industrial Group in collaboration with the Iranian state [169]. The vaccine utilizes an inactivated virus based. The vaccine administration follows a two-dose intervention, intramuscular route (each dose is 28 days apart). The vaccine showed clinical efficacy in pre-clinical studies in mice, rabbits, and non-human primates [170,171]. The company began a phase-I trial, which is approved by the Iranian government (IRCT20201202049567N1). The vaccine has been authorized for human use in emergency conditions in Middle East countries. Recent studies have reported an efficacy of 93.8% with this vaccine [172].

### 5.4. Recombinant Protein Subunit Vaccines

Protein subunit vaccines uses a fragment of specific proteins, which is packed into Nano-based particles [173,174]. These vaccines are very safe and showed promising results in combating the SARS-CoV-2. Several vaccines of this type are in the pre-clinical trials and using different protein subunits for boosting the immune responses [173,174]. According to a few reports, these subunits showed poor immunogenic properties and they require the adjuvants for their therapeutic actions. However, these vaccines were able to induce poorer stimulation of CD8+ T cells and CD4+ T-cell activation, and also specifically neutralize the specific antibody responses.

#### 5.4.1. EpiVacCorona Vaccine (EVCV)

EpiVacCorona Vaccine is also called as (EVCV) is manufactured by the Vector Institute, which is a Russian biological experimental canter. These vaccines utilize the fragments of synthetic viral peptides, which reflect those of SARS-CoV-2-like antigens [175]. EVCV is administered in two-dose regimen by an intra muscular injection and is approved for the individuals above age of 18 years as well as below 60 years [176]. A phase 1/2 trial conducted in Russia, which is mainly evaluated the EpiVac Vaccine efficacy in 100 individuals (NCT04527575).

#### 5.4.2. ZF2001 ((RBD Dimer) ZIFIVAX)

ZF2001 vaccine is developed by the collaboration with the Chinese Anhui Zhifei Longcom and the Academy of Military Medical Sciences located in China. This vaccine utilizes a specific section of spike protein of receptor-binding domain (RBD) and is combined with an adjuvant [174]. According to recent studies, the vaccine is safe and provided immense immunological responses. The vaccine administration follows three-dose interventions of 4 weeks apart by intramuscular route of administration. Although the vaccine is currently in phase-3 clinical trials, it has been approved for the emergency purpose in China and Uzbekistan [177]. Phase 1/2 trial results, published in The LANCET journal showed that the vaccine was well-tolerated and generated immune response in participants [177]. Data from a pre-print posted on 2 February 2021 showed that the vaccine neutralizes B.1.351 (Beta) variant of SARS-CoV-2 [178]. In addition, the vaccine candidate was found effective against the B.1.617.2 (Delta) variant. 

#### 5.4.3. Abdala Vaccine (CIGB 66)

Abdala, also known as CIGB-66, is a COVID-19 vaccine developed by the Center for Genetic Engineering and Biotechnology located in Cuba. This vaccine was developed based on the recombinant spike RBD protein produced in a yeast called Pichia pastoris. Its phase I/II clinical trial, initiated in the first week of December, 2020 [179] and the phase III trial was started in the end of March 2021 [179] with an efficacy of 92.28% [180]. The vaccine was approved for emergency use in Cuba on 9th July 2021 [181]. Data show that the Abdala vaccine was more than 90% effective against severity and death [180].

#### 5.4.4. Soberana Vaccine

The Soberana vaccine is manufactured by the Finlay Institute of Vaccines, Cuba and Pasteur Institute, Iran. SOBERANA 02 is a protein subunit conjugate vaccine in which RBD is conjugated to tetanus toxoid (TT). The vaccine is administered as a two-dose intervention with the second dose provided after 28 days of first dose. According to a recent report, the vaccine showed an efficacy of 71% after two-dose administration, but, when combined with a booster dose of Soberana plus it has yielded 92.4% efficacy [182]. Soberana 2 was evaluated in 40 individuals in phase-1 (RPCEC00000340) trial. Currently, a phase 2 trial is being conducted in 910 individuals.

#### 5.4.5. MVC COVID (MVC-COV1901)

Medigen, also known as MVC-COVID-19 (MVC-COV1901), is manufactured by the Medigen Vaccine Biologics Corporation in Taiwan in collaboration with Dynavax Technologies and National Institute of Health USA. The vaccine is developed using protein sub-unit technologies and contain a recombinant spike protein (S-2P) and am adjuvant with specific CpG-1018 supplied with a Dynavax [183]. The vaccine activates robust immune responses in individuals and a humoral immune response by binding of IgG to S-protein. In addition, the vaccine triggers cellular immune responses by promoting IFN-ϒ and IL-4 secreting T-cells [184]. Results from the phase-II trial from Taiwan and Vietnam showed that the vaccine had good safety profile with no severe adverse events reported [185]. Phase-I results demonstrated that the vaccine had a robust immune response [184].

#### 5.4.6. Zycov-D

ZycoV-D, also called as a plasmid vaccine, is a plasmid DNA intradermal vaccine candidate, manufactured by the Zydus Cadilla Healthcare Private Limited, India. Zycov inhibits the viral entry into the host cells thereby reduces its spread. Dose of administration was for 56 days apart in three-dose regimen [186]. A phase 1/2 trial conducted in 1000 healthy individuals for dose escalation studies (CTRI/2020/07/026352) reported that Zycov is effective (66.6% efficacy) [186].

#### 5.4.7. Spikogen

Spikogen vaccine, also called as Cova-19, developed by Australia Vaxine Pvt Ltd. together with Iranian biotech company Cinnagen. The vaccine works on the basis of a monovalent recombinant protein [187]. It has been approved for emergency use in Iran. A phase-I randomized; placebo-controlled trial was conducted on 40 individuals aged between 18–65 years. The study participants had received two doses of Spikogen 25 µg and Advax-2 15mg as an adjuvant 3 weeks apart (NCT04453852).

#### 5.4.8. Fakhravac

Fakhravac, also named MIVAC, comes under the inactivated vaccine category. It was developed by the Stem Cell Technology Research Center in Iran. Based on the results of phase-I trial conducted in Iran with 135 individuals (IRCT20210206050259N1), and a phase-II trial with 500 participants (IRCT20210206050259N3), on 9 September 2021 the government of Iran authorized the vaccine for emergency conditions; however, it was discontinued from the market due to the lack of demand (https://en.irct.ir/trial/56027 (accessed on 21 June 2022)).

#### 5.4.9. Nuvaxovid

Nuvaxovid, also named as Covovax, was manufactured by Novovax (a Maryland, USA-based company) in collaboration with the GSK and Sanofi. The vaccine is prepared by attaching the viral proteins on to nanoparticles/microscopic particles [188]. Covovax is administered by intramuscular injection, following the two-dose regimen for 21 days apart. Covovax has been shown to induce a strong antibody response while activating T-cells [189]. Nuvaxovid is stable at refrigerator temperatures (2 °C–8 °C).

A phase-III trial conducted in UK showed that the Novavax is effective against different variants of SARS-CoV-2 [190]. It showed 89.7% overall efficacy and 86.3% against B.1.1.7 (ALPHA) variant, 96.4% for other non-B.1.1.7 variants [190]. Novavax showed an efficacy of 96.4% in phase-III trial in UK against the SARS-CoV-2, 55.4% efficacy against B.1.351 (BETA VARIANT) [191]. A phase-III trial showed about 90.4% overall efficacy, and 92.6% efficacy against SARS-CoV-2 [191]. Another clinical study has shown that Novavax is well tolerated and triggered a robust immune response [192]. Recent reports have shown an overall efficacy against the B.1.351 variant. An efficacy of 60.1% was reported in HIV negative individuals [18]. A phase 1/2 trial showed that patients who received the vaccine developed antibody responses at multiple doses [193].

#### 5.4.10. Turkovac

Turkovac, also named ERUCOV-VAC, was developed by health institutes of Turkey in collaboration with TC Erciyes university. This vaccine was developed as an inactivated COVID-19 vaccine. A phase-I trial in 44 participants (NCT04691947), and a phase-II trial in 250 individuals (NCT04824391) was conducted to test its safety and efficacy. On December 22, 2021 Turkey’s president announced that Turkovac is approved for emergency use in the country.

#### 5.4.11. Carbovax

Carbovax was developed by the Biological E company, India. Carbovax is an adjuvanted protein subunit vaccine. A phase 1/2 trial, which was conducted in 360 healthy participants in India (CTRI/2020/11/029032) [194]. Drug Controller General of India has approved a phase 2/3 trial of this vaccine in children and adolescents of age group between 5–18 years. This vaccine is approved for administration in Botswana.

#### 5.4.12. Covifenz

Covifenz vaccine was developed by Medicago, a Canadian company. This vaccine is made up of SARS-CoV-2 spike recombinant (adjuvant) protein virus-like particle and is administered in two doses (21 days apart). This vaccine is approved for emergency use in Canada. A phase-I trial was conducted in 180 participants of age group between 18–55 years using a dose of 3.75 µg, 7.5 µg and 15 µg (NCT04450004). Recent reports have stated that the vaccine is effective against SARS-CoV-2 and its multiple variants (69.5% and 78.8% effective against moderate to severe disease) [195].

#### 5.4.13. VLA2001

This vaccine was developed by the Valneva Company in France. It works through the inactivated vaccine protocol. VLA2001 contains CpG 1018 as an adjuvant, provided by the Dynavax. This vaccine was approved for the emergency use in Bahrain and in United Kingdom [196]. A phase-III trial, referred as COV-COMPARE trial, was conducted in about 4000 participants to test the efficacy of Valneva’s vaccine with AstraZeneca’s Vaxzevria (NCT04864561). Recent reports have shown that the vaccine is well tolerated in participants. Further studies are needed to test its efficacy against other SARS-CoV-2 variants.

#### 5.4.14. Noora

Noora vaccine was developed by the Baqiyatallah University of Medical Sciences in Iran working under the supervision of the Islamic Revolution Guards Crops (IRGC). This is a protein vaccine administered in three doses, with a second dose on the 21st day and the third dose on the 35th day. A phase 2 trial of Noora is underway (IRCT20210620051639N3).

**Table 6:** List of vaccines authorized for combating the SARS CoV-2 and their reported efficacies.

S. No	Vaccine Name	Type of Vaccine	Primary Developers	Vaccine Efficacy on SARS-CoV-2 Wild Type or Variants	Primary End Point or Outcome	Number of Doses of Vaccination
1	BNT162b2 (Comirnaty)	mRNA-based vaccine	Pfizer, BioNTech; Fosun Pharma	SARS-CoV-2: 95% [76] Omicron: 70% [22]	Safety over a median of 2 months was similar to that of other viral vaccines	Two doses
2	mRNA-1273 (Spikevax)	mRNA-based vaccine	Moderna, BARDA, NIAID	SARS-CoV-2: 94.1% [130] Omicron: 85% [132]	The primary end point was prevention of COVID-19 illness with onset at least 14 days after the second injection in participants who had not previously been infected with SARS-CoV-2.	Two doses
3	AstraZeneca (AZD1222 also known as Vaxzevria and Covishield	Adenovirus vaccine	BARDA, OWS	74% (Overall) and an efficacy of 83.5% in participants age 65 years and older [140]	Preventing the onset of symptomatic and severe coronavirus disease 2019 (COVID-19) 15 days or more after the second dose in adults, including older adults	Two doses
4	Sputnik V (Gam-COVID-Vac)	Recombinant adenovirus vaccine (rAd26 and rAd5)	Gamaleya Research Institute, Acellena Contract Drug Research and Development	91.6% [143]	The primary outcome was the proportion of participants with PCR-confirmed COVID-19 from day 21 after receiving the first dose.	Two doses
5	Janssen (JNJ-78436735; Ad26.COV2.S)	Non-replicating viral vector	Janssen Vaccines (Johnson & Johnson)	52.9% against moderate or severe-to-critical COVID-19 and 41.7% against any infection [150]	The primary end points were vaccine efficacy against moderate to severe–critical COVID-19 with onset at least 14 days after administration	Single dose
6	CoronaVac	Inactivated vaccine (formalin with alum adjuvant)	Sinovac	65.9% for the prevention of COVID-19 and 87.5% for the prevention of hospitalization, 90.3% for the prevention of ICU admission, and 86.3% for the prevention of COVID-19–related death [161]	Estimated the change in the hazard ratio associated with partial immunization (≥14 days after receipt of the first dose and before receipt of the second dose) and full immunization (≥14 days after receipt of the second dose)	Both Single and Double dose
7	BBIBP-CorV (Vero Cells) also called as Covilo	Inactivated vaccine	Beijing Institute of Biological Products; China National Pharmaceutical Group (Sinopharm)	78.1% [155]	Primary outcome was efficacy against laboratory-confirmed symptomatic COVID-19 14 days following a second vaccine dose among participants who had no virologic evidence of SARS-CoV-2 infection at randomization. The secondary outcome was efficacy against severe COVID-19.	Two Doses
8	Convidicea (PakVac, Ad5-nCoV)	Recombinant vaccine (adenovirus type 5 vector)	CanSino Biologics	57.5% [148]	The primary efficacy objective evaluated Ad5-nCoV in preventing symptomatic, PCR-confirmed COVID-19 infection occurring at least 28 days after vaccination in all participants who were at least 28 days postvaccination	Single dose
9	Covaxin (BBV152)	Inactivated vaccine	Bharat Biotech, ICMR; Ocugen; ViroVax	77.8% [165]	The primary outcome was the efficacy of the BBV152 vaccine in preventing a first occurrence of laboratory-confirmed (RT-PCR-positive) symptomatic COVID-19 (any severity), occurring at least 14 days after the second dose in the per-protocol population.	Two Doses
10	WIBP-CorV	Inactivated vaccine	Wuhan Institute of Biological Products; China National Pharmaceutical Group (Sinopharm)	72.8% [155]	Primary outcome was efficacy against laboratory-confirmed symptomatic COVID-19 14 days following a second vaccine dose among participants who had no virologic evidence of SARS-CoV-2 infection at randomization. The secondary outcome was efficacy against severe COVID-19.	Two Doses
11	Zycov-D	DNA vaccine (plasmid)	Zydus Cadila	66.6% [186]	The primary outcome was the number of participants with first occurrence of symptomatic RT-PCR-positive COVID-19 28 days after the third dose	Three doses
12	Nuvaxovid (Covovax in India; previously NVX-CoV2373)	Recombinant nanoparticle vaccine	Novavax CEPI, Serum Institute of India	89.7% [190]	The primary efficacy end point was virologically confirmed mild, moderate, or severe SARS-CoV-2 infection with an onset at least 7 days after the second injection in participants who were serologically negative at baseline.	Two doses
13	Covifenz (CoVLP)	Plant-based adjuvant vaccine	Medicago; GSK; Dynavax	78.8% [195]	The primary objective of the trial was to determine the efficacy of the CoVLP+AS03 vaccine in preventing symptomatic coronavirus disease 2019 (COVID-19) beginning at least 7 days after the second injection	Two Doses
14	Soberana 02/Soberana Plus	Conjugate vaccine	Finlay Institute of Vaccines; Pasteur Institute	92.4% [182]	Study endpoints are vaccine efficacy (VE) evaluated through confirmed symptomatic COVID-19 and safety	Three doses

## 6. Discussion and Conclusions

Despite vaccination against COVID-19, cases and mortality continue worldwide, largely due to the emergence of new variants. These variants arise from mutations. Mutations in the virus not only influences the virulence, transmissibility, and pathogenesis of virus but also create major hurdles in the disease diagnosis, treatment as well as vaccine development. Mutations are characteristic feature of all viruses; however, the rate of mutations in RNA viruses is more than five-fold when compared to DNA viruses. In the process of evolution, viruses mutate continually, and new variants are expected to appear and disappear randomly and sometimes persist where natural selection decides the destiny of the newly created variant. Generally, mutations, which offer negative advantage with respect to transmission, viral replication, or immunity escape, will consequently reduce the viral efficiency.

Since spike protein of SARS-CoV-2 is one of the primary targets in vaccine design, mutations in this gene regions can reduce vaccines’ effectiveness against the virus. Hence, mutations generated in the emerging variants should be monitored. Currently, Delta and Omicron are the two variants of concern, which are reported to have increased transmissibility, virulence, and/or decreased diagnostic, therapeutic, or vaccine efficacy. Compared with earlier variants, Omicron has more than 60 mutations, with ~30 mutations in the S protein and almost 15 mutations in the RBD, resulting in rapid attachment to human cells and escape immunity. It is presumed that, when compared to the Delta variant, Omicron cases will surge dramatically due to high disease transmission rate, but will be less severe among hospitalized patients. Due to the 2–3 times higher transmission rates compared to the Delta variant, Omicron is predicted to infect several people, because of which the Delta variant might fade away gradually, which might be the ending phase of a pandemic or the starting phase of an endemic.

It was assumed that as there are several similarities between the Omicron variant and pandemics from the history, such as low virulence, high transmission rate, and typical symptoms, that Omicron might decline in the same way through vaccination. However, recent studies have shown that Omicron might not be the end of the pandemic as a super variant, Delmicron (Deltacron), a combination of Delta and Omicron variants recombinant, is being identified in some places around the world. Demicron might exert pathogenicity, which is similar to Delta variant and a transmissibility rate, similar to Omicron, and might become more severe due to a mixture of both VOCs. However, to date, not many Demicron variant cases are reported.

These new variants might penetrate and might infect unvaccinated or vaccinated individuals by facilitating immune escape, which can prompt these individuals to severe disease or death and increase the chances of new variants in future. Nevertheless, several studies have reported and suggested that vaccination is still effective against the current circulating variants and can protect against severe to moderate disease consequences. As of 13 May 2022, a total of 354 COVID-19 vaccine candidates are in various stages of development, with 156 in clinical and 198 in preclinical development. Available vaccines are showing promising results against both wildtype and mutant variants. While it is difficult to predict the next VOC, it is feasible to learn from experiences and challenges to better manage with existing situations and avoid the spread of viruses. To reduce the risk of new and potentially more deleterious variants emerging, and considering existing scientific evidence that suggests benefits of vaccination against SARS-CoV-2, health authorities may focus on vaccinating eligible individuals as expeditiously as possible. We therefore suggest that vaccination strategies be continued. Vaccination may even be beneficial in situations where all available vaccines are less effective against certain variants of the virus. In summary, vaccinating individuals will help to better fight against SARS-CoV-2 and its variants.

## Figures and Tables

**Table 1:** Genes and proteins expressed by SARS-CoV-2.

Number	Gene	Nucleotide Location	Protein	Amino Acids
**1**	ORF1a	266–13,483	Polyprotein 1a	4405
**1**	ORF1ab	266–21,555	Polyprotein 1ab	7096
**2**	ORF2	21,563–25,384	Surface glycoprotein or Spike (S protein)	1273
**3**	ORF3a	25,393–26,220	ORF3a protein	275
**4**	ORF4	26,245–26,472	Envelope protein (E protein)	75
**5**	ORF5	26,523–27,191	Membrane glycoprotein (M protein)	222
**6**	ORF6	27,202–27,387	ORF 6 protein	61
**7**	ORF7a	27,394–27,759	ORF 7a protein	121
**8**	ORF7b	27,756–27,887	ORF 7b protein	43
**9**	ORF8	27,894–28,259	ORF 8 protein	121
**10**	ORF9	28,274–29,533	Nucleocapsid phosphoprotein (N protein)	419
**11**	ORF10	29,558–29,674	ORF10 protein	38

**Table 2:** Structural and functional details of NSPs of SARS-CoV-2.

Name	Function	Nucleotide Location	Amino Acids
NSP1	Host mRNA degradation and translation inhibition	266–805	180
NSP2	Unknown	806–2719	638
NSP3	Polyprotein processing, de-ADP ribosylation, Deubiquitinating, Interferon antagonist, DMV (double-membrane vesicle) formation	2720–8554	1945
NSP4	DMV formation	8555–10,054	500
NSP5	Polyprotein processing, Inhibition of interferon signaling	10,055–10,972	306
NSP6	DMV formation	10,973–11,842	290
NSP7	Cofactor for RNA-dependent RNA polymerase	11,843–12,091	83
NSP8	primase or 3′-terminal adenylyl transferase, cofactor for RNA-dependent RNA polymerase	12,092–12,685	198
NSP9	Binding of single-stranded RNA	12,686–13,024	113
NSP10	Cofactor for nsp14 and 16	13,025–13,441	139
NSP11	Unknown	13,442–13,480	13
NSP12	RNA-dependent RNA polymerase, Nucleotidyl transferase	13,442–16,236	932
NSP13	Helicase, RNA 5′ triphosphatase	16,237–18,039	601
NSP14	3′ to 5′ exoribonuclease, Proofreading, RNA cap formation, Guanosine N7-methyltransferase	18,040–19,620	527
NSP15	Endoribonuclease, evasion of immune response	19,621–20,658	346
NSP16	RNA cap formation, Ribose 2′ O-methyltransferase	20,659–21,552	298

**Table 3:** Current circulating VOC and mutations reported.

S. No.	WHO Designation	Pango Lineage	Nextstrain Clade	GISAID Clade	Mutations Reported
**1**	Omicron	B.1.1.529	21K, 21L, 21M	GR/484A	**ORF1a**—T3255I, P3395H, P314L, I1566V **S**—G142D, G339D, S373P, S375F, K417N, N440K, S477N, T478K, E484A, Q493R, Q498R, N501Y, Y505H, D614G, H655Y, N679K, P681H, N764K, D796Y, Q954H, N969K **E**—T9I **M**—Q19E, A63T **ORF8**—S84L **N**—P13L, del131/133, R203K, G204R

**Table 4:** List of current circulating Variants Being Monitored (VBM) and mutations reported.

S. No.	WHO Designation	Pango Lineage	Nextstrain Clade	GISAID Clade	Mutations Reported
**1**	Alpha	B.1.1.7	20I (V1)	GRY	**ORF1a**—T1001I, A1708D, I2230T, del3675/3677 **ORF1b**—P314L **S**—del69/70, del144/145, N501Y, A570D, D614G, P681H, T716I, S982A, D1118H **ORF8**—Q27 *, R52I, Y73C, S84L **N**—D3L, R203K, G204R, S235F
2	Beta	B.1.351	20H (V2)	GH/501Y.V2	**ORF1a**—T265I, K1655N, K3353, del3675/3677 **ORF1b**—P314L **S**—D80A, D215G, del241/243, K417N, E484K, N501Y, D614G, A701V **ORF3a**—Q57H, S171L, **E**—P71L **ORF8**—S84L **N**—T205I
**3**	Gamma	P.1	20J (V3)	GR/501Y.V3	**ORF1a**—S1188L, K1795Q, del3675/3677 **ORF1b**—P314L, E1264D **S**—L18F, T20N, P26S, D138Y, R190S, K417T, E484K, N501Y, D614G, H655Y, T1027I, V1176F **ORF3a**—S253P **ORF8**—S253P, E92K **N**—P80R, R203K, G204R
4	Delta	B.1.617.2	21A, 21I, 21J	G/478K.V1	**ORF1a**—A1306S, P2046L, P2287S, V2930L, T3255I, T3646A **ORF1b**—P314L, G662S, P1000L, A1918V **S**—T19R, E156G, del157/158, L452R, T478K, D614G, P681R, D950N **ORF3a**—S26L **M**—I82T **ORF7a**—V82A, T120I **ORF7b**—T40I **ORF8**—S84L, del119/120 **N**—D63G, R203M, G215C, D377Y
**5**	Epsilon	B.1.427 B.1.429	21C	GH/452R.V1	ORF1a—T265I ORF1b—P314L, D1183Y S—S13I, W152C, L452R, D614G ORF3a—Q57H, ORF8—S84L N—T205I
6	Eta	B.1.525	21D	G/484K.V3	ORF1a—T2007I, del3675/3677 ORF1b—P314F S—Q52R, A67V, del69/70, del144/144, E484K, D614G, Q677H, F888L E—L21F M—I82F ORF6—del2/3 ORF8—S84L N—S2Y, del3/3, A12G, T205I
7	Lota	B.1.526	21F	GH/253G.V1	**ORF1a**—T265I, L3201P, del3675/3677 **ORF1b**—P314L, Q1011H **S**—L5F, T95I, D253G, D614G **ORF3a**—P42L, Q57H **ORF8**—T11I, S84L
8	Kappa	B.1.617.1	21B	G/452R.V3	**ORF1a**—T1567I, T3646A **ORF1b**—P314L, M1352I, K2310R **S**—L452R, E484Q, D614G, P681R, Q1071H **ORF3a**—S26L **ORF7a**—V82A **ORF8**—S84L **N**—R203M, D377Y
9	Mu	B.1.621	21H	GH	**ORF1a**—T1055A, T1538I, T3255I, Q3729R **ORF1b**—P314L, P1342S **S**—T95I, Y145N, R346K, E484K, N501Y, D614G, P681H, D950N **ORF3a**—Q57H, del256/257 **ORF8**—T11K, P38S, S67F, S84L **N**—T205I
10	Zeta	P.2	20B/S.484K	GR/484K.V2	**ORF1a**—L3468V, L3930F **ORF1b**—P314L **S**—E484K, D614G, V1176F **ORF8**—S84L **N**—A119S, R203K, G204R, M234I

* Indicates stop mutation.

**Table 5:** Tabular representation of vaccines in clinical development.

S. No.	Platform	Vaccine Candidate	Number
1	PS	Protein subunit	52
2	VVnr	Viral Vector (non-replicating)	21
3	DNA	DNA	16
4	IV	Inactivated Virus	21
5	RNA	RNA	30
6	VVr	Viral Vector (replicating)	4
7	VLP	Virus Like Particle	6
8	VVr + APC	VVr + Antigen Presenting Cell	2
9	LAV	Live Attenuated Virus	2
10	VVnr + APC	VVnr + Antigen Presenting Cell	1
11	BacAg-SpV	Bacterial antigen-spore expression vector	1

## Data Availability

Not applicable.
